# Potential of Lipid Biosynthesis under Heterotrophy in the Marine Diatom *Cyclotella cryptica*

**DOI:** 10.1021/acssuschemeng.3c02542

**Published:** 2023-12-04

**Authors:** Salvatore Morra, Mariamichela Lanzilli, Angela Grazioso, Adelaide Cupo, Simone Landi, Genoveffa Nuzzo, Daniela Castiglia, Carmela Gallo, Emiliano Manzo, Angelo Fontana, Giuliana d’Ippolito

**Affiliations:** † National Research Council (CNR), Institute of Biomolecular Chemistry (ICB), Via Campi Flegrei 34, 80078 Pozzuoli, Naples, Italy; ‡ Department of Biology, University of Naples “Federico II”, Via Cinthia, I-80126 Napoli, Italy

**Keywords:** Green chemistry, Bioprocess sustainability, Microalgae, Biodiesel, Biofuels, Fatty acids

## Abstract

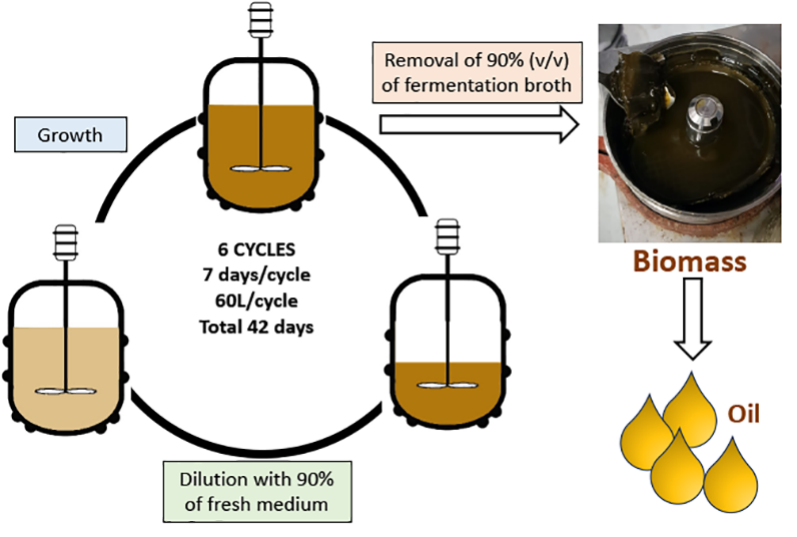

Despite the theoretical high productivity, microalgae-based oil production is not economically sustainable due to the high cost of photoautotrophic cultures. Heterotrophic growth is a suitable economic alternative to overcoming light dependence and climatic/geographic fluctuations. Here we report data about growth performance, biomass production, and lipid composition of the marine diatom *Cyclotella cryptica*, chosen as a model strain for biodiesel production in heterothrophy. A repeated-batch process of heterotrophic cultivation has also been investigated to assess the robustness and phenotypic stability. The process consisting of six constant cycle repetitions was carried out for 42 days and led to an average dry biomass production of 1.5 ± 0.1 g L^–1^ of which 20% lipids composed of 60% triglycerides, 20% phospholipids. and 20% glycolipids. The major fatty acids were C16:0 (∼26%), C16:1 ω-7 (∼57%), and C20:5 ω-3 (∼12%), with a significant reduction in the unsaturated fatty acids in comparison to other microalgae grown in heterotrophy. Fatty acids were differently distributed among the glycerolipid classes, and the lipid composition was used to compare the potential properties of *C. cryptica* oil with traditional vegetable biofuels.

## Abstract 2

Batch and repeated-batch processes for evaluating lipid composition and biodiesel production by heterotrophic cultivation of the marine diatom *Cyclotella cryptica*.

## Introduction

Biofuels represent a low-carbon alternative to fossil fuels because they can help reduce greenhouse gas emissions and the associated impact of transportation on climate change.^1^ Biodiesel is a mixture of monoalkyl esters of long-chain fatty acids that are a renewable source of energy with almost net-zero carbon emissions. Currently, biodiesel is manufactured by vegetable oils from conventional feedstocks which only partially satisfy existing demand and compete in the use of agricultural land for food production.^2−4^ Microalgae have been suggested to be a sustainable alternative source of renewable fuel due to their high productivity and low land requirements to conventional crops.^5−8^ In addition, microalgae support more sustainable biofuel production because they can adapt to variable grow conditions, allowing cultivation in harsh environments unsuitable for other existing biofuel feedstocks.^9,10^ Facilities dedicated to the photosynthetic massive production of microalgae traditionally involve the use of open ponds or photobioreactors. In spite of many advantages, photoautotrophy can be limited by light availability in large-scale cultivation and is susceptible to changes in climate and temperature.^11,12^ A valid alternative to autotrophic culture is to exploit the ability of microalgae to grow in heterotrophic conditions.^13,14^ Heterotrophic cultivation is carried out in the dark using exogenous sources of organic carbon to support cell growth and energy needs.^15−18^ This process enables obtaining high cell density and lipid productivity and circumvents light-dependent limitations related to photosynthetic efficiency, manufacturing, and seasonality of biomass harvest, offering the possibility to achieve high cell density. Heterotrophic cultures can produce 50–100 g/L of dry biomass, well above the maximum 30 g/L reported in photoautotrophy, while maintaining a lipid content from 40% to 73% of dry cell weight.^19,20^ On the other hand, heterotrophic cultivation still suffers from numerous bottlenecks including (1) low availability of wild species capable of growing heterotrophically (e.g., genera *Chlorella*, *Galdieria*, *Nitzchia*, *Crypthecodinium*, and *Neochloris*), (2) growth inhibition at high concentrations of organic substrates used as carbon source, (3) prices of organic substrates which can account for over 65% of total operating costs, and (4) risks of bacterial contamination in media rich in organic carbon.^21−25^ Currently, the main challenge is the identification of robust microalgae species capable of ensuring a high conversion rate of sugar-to-biomass, a high content of oil with optimal compositions, a fermentation processe on biowaste rich in sugars (e.g., repeated batch, fed-batch, perfusion).^18,25−27^

Diatoms include over 200,000 estimated species, widely distributed in diverse natural habitats where they form large biomasses during seasonal blooms that sustain the aquatic food web and primary production on Earth.^28,29^ Due to their resilience, metabolic plasticity, and capacity of accumulating lipids to a greater extent than other microalgae, diatoms have been often explored as biological platforms for the production of oil and valuable products.^30−32^ However, studies related to heterotrophic cultivation are quite uncommon and mainly focus on the optimization for the production of eicosapentaenoic acid (EPA) and docosahexaenoic acid (DHA).^26,33−35^

Recently, we reported that the marine diatom *Cyclotella cryptica* is a promising source of EPA in the dark.^34^ Here we investigate the effect of this condition on the total lipid composition and productivity by batch and repeated-batch processes. In order to test the potential of *C. cryptica* for future commercial applications, the study also estimates the quality of biodiesel produced from heterotrophic cultivation of the diatom.

## Experimental Section

### General

All solvents and standards were purchased from Sigma-Aldrich (Milan, Italy). ^1^H NMR spectra were recorded by using a Bruker DRX 600 spectrometer (Bruker, Milan, Italy) equipped with an inverse TCI CryoProbe. Spectra were recorded with 32 K time domain data points, 90° pulse, 32 K spectral size, 14 ppm spectral width (8417.5 Hz), and 0.6 Hz of line broadening for the exponential decay function. Peak integration, ERETIC measurements (Electronic REference To access In vivo Concentrations), and spectrum calibration were performed using specific subroutines of the Bruker Top-Spin 3.1 software.

### Strain and Culture Conditions

*Cyclotella cryptica* (CCMP 331) was purchased from the National Center for Marine Algae and Microbiota (NCMA, USA https://ncma.bigelow.org/) and was maintained in flasks at 20 ± 2 °C in prefiltered sterile (0.22 μm) f/2 medium. Artificial illumination (200 μmol photons m^–2^ s^–1^) was guaranteed by fluorescent tubes (OSRAM 965, Germany) using a light:dark photoperiod 14:10 h.^36^ In heterotrophic conditions, cultures in a 10 L polycarbonate carboy were incubated in a dark chamber at 20 ± 2 °C and gently bubbled with sterile air. Cells were grown in a prefiltered sterile (0.22 μm) f/2 medium containing 4g L^–1^ glucose (133 μmole carbon L^–1^), 0.370 g L^–1^ NaNO_3_ (4.4 μmole nitrogen L^–1^, C/N = 30), 0.035g L^–1^ NaH_2_PO_4_·H_2_O (0.25 μmole posphorous L^–1^, C/P = 525), 0.38 g L^–1^ Na_2_SiO_3_·9H_2_O (1.33 μmole silicon L^–1^, C/Si = 100). Monitoring of cell growth was carried out by a microscope (Axio VertA1, Carl Zeiss, 20× objective) and a Bürker counting chamber (depth 0.100 mm, Merck, Leuven, Belgium).

### Growth Rate (μ) and Duplication Time (*t*_*d*_) Calculation

Growth rate μ (expressed as divisions day^–1^) and doubling time *t*_*d*_ (time required to achieve a doubling of the number of viable cells) were calculated in agreement with Cupo et al.,^34^ as follows:
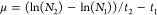
1
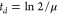
2where *N*_1_ and *N*_2_ represent the number of cells (cells mL^–1^) at time 1 (*t*_1_) and time 2 (*t*_2_) at the extremes of the linear phase.

### Repeated Batch Cultures

Seed culture expansion was performed in several repeated batch cultures. Cells from flasks were grown in one 10 L carboy from an initial concentration of 2.5 × 10^5^ cells mL^–1^ for 7 days (starter cycle). Aliquots of these cultures were then inoculated in six 10 L carboys at an initial concentration of 2.5 × 10^5^ cells mL^–1^. The first cycle (I) was performed by transferring 10% (v/v) of the seed culture (1 L) into 9 L of fresh medium to obtain a final culture volume of 10 L. The subsequent consecutive cycles II–VI were conducted following a similar experimental scheme. In each cycle, six 10 L carboys were inoculated with 1/10 of the cells deriving from the previous cycle and grown simultaneously for 7 days. The entire repeated batch process was run for six cycles over 42 days.

### Biomass Content

Cells (500 mL) were harvested by centrifugation (Allegra X-12R, Beckman Coulter Inc., Palo Alto, CA, USA) at 2300 rpm for 10 min at 4 °C, using a swing-out rotor). The supernatant was filtered through a 0.22 μm filter and stored at −20 °C for the analysis of glucose consumption. Pellets were frozen at −80 °C and lyophilized with a MicroModulyo 230 (Thermo Electron Corporation, Milford, MA, USA). Dry biomass was expressed as mg L^–1^ culture and calculated by weighing lyophilized biomass. Biomass productivity was calculated in agreement with d’Ippolito et al.^32^

### Glucose Consumption

Glucose concentration was measured by proton NMR using the ERETIC method.^37^ For the analysis, 50 μL of deuterium water (D_2_O) was added to 650 μL of culture medium in a 5 mm NMR tube. Protons at 3.24 ppm of C-2 of ß-glucose were used for the area integration. The equilibrium between 36% α-anomer and 64% β-anomer of glucose in water was considered in the calculations. Reference was a standard solution of glucose, 1 mg mL^–1^ (5.5 μmol L^–1^).

### Lipid Extraction

Lipid extraction was performed following methyl *tert*-butyl ether (MTBE) method.^37^ 4,4′-Dihydroxybenzophenone (DHBP) (1 mg mL^–1^) was used as internal standard. Briefly, a dry cell pellet (50 mg) was suspended in 400 μL of MeOH and 500 μL of DHBP solution. After vortexing, MTBE (3 mL) was added, and the sample was shaken at room temperature for 1 h. Then, 750 μL of water was added, and the suspension was shaken for 10 min at room temperature prior to recovery of the organic phase (upper phase) by centrifugation at 1000 rpm for 10 min. Water (750 μL) was added to the residual aqueous phase and re-extracted with 1 mL of MTBE by the same procedure. Organic phases were combined and dried under a nitrogen flow. The final extract was weighed to gravimetrically estimate lipid content (mg L^–1^ culture), lipid productivity (mg L^–1^ culture day^–1^), and lipid percentage.

### NMR Analysis of Lipid Extracts

Crude organic extracts of microalgae were dissolved in 700 μL CDCl_3_/CD_3_OD 1:1 (v/v) and transferred to a 5 mm NMR tube for ^1^H NMR analysis with ERETIC method.^37^ The reference signal was calibrated on the doublet at δ 6.90 of 4,4′-dihydroxybenzophenone (DHBP) (2.23 μmol in 700 μL of CDCl_3_/CD_3_OD 1:1). Quantitative analysis was based on the integration of the following signals: doublet at 4.90 ppm (*J* = 3.5 Hz) due to the anomeric proton of galactose in digalactosyldiacylglycerols (DGDG), doublet at 4.80 ppm (*J* = 3.7 Hz) due to the anomeric proton of sulfoquinovose in sulfoquinovosyldiacylglycerols (SQDG), multiplet between 4.53 and 4.38 ppm due to methylene protons of glycerol in phospholipids (PL) and glycolipids (GL), double doublet at 4.34 ppm (*J* = 4.0, 12.0 Hz) due to the methylene protons of glycerol in triacylglycerols (TAG), and doublet at 3.88 ppm (*J* = 3.0 Hz) due to the methine proton at C4 of galactose of monogalactosyldiacylglycerols (MGDG). The diagnostic signals in the region between 2.38 and 2.28 ppm were used to assess μmol of total fatty acids (TFA). The amount of each class was expressed as mole number and corrected for the standard recovery.

### GCMS Analysis of Lipid Extracts

The total fatty acid composition was determined by GCMS on the corresponding fatty acid methyl esters (FAME). FAME were obtained by saponification of the lipid extracts with sodium carbonate (Na_2_CO_3_) in methanol at 40 °C for 4 h.^34^ The reaction mixture was diluted with milli-Q water (to completely dissolve Na_2_CO_3_), neutralized with 1 M HCl, and extracted with *n*-hexane three times. Combined organic extracts were dried under a nitrogen stream, dissolved in MeOH at a final concentration of 1 μg μL^–1^, and analyzed by GSMS (Thermo Focus GC Polaris Q instrument) equipped with a 5% diphenyl column. GCMS parameters included 70 eV for the ion-trap, 210 °C for the injector, and 280 °C for the transfer line. FAME elution was performed according to the following gradient of temperature: 160 °C for 3 min followed by a first increase of 3 °C/min up to 260 °C and a second increase of 30 °C/min up to 310 °C. Finally, the temperature was kept at 310 °C for 7 min. FAME have been identified by comparison of retention time and mass spectra with a standard mixture (Marine source analytical standards, Sigma-Aldrich). Fatty acid (FA) content of each chemical species was expressed as % of total fatty acids, according to the formula:
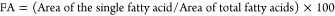
3

### Calculation of Fuel Properties from Fatty Acid Profiles

Estimation of biodiesel properties was derived by the empirical formulas proposed by Islam and Patel.^39,40^ Long-chain saturation factor (LCSF) was calculated based on the equation
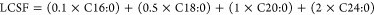
4

The cold filter plugging point (CFPP, °C) was calculated according to the equation:
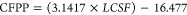
5

Oxidation stability (OS), expressed in hours (h), is influenced by the age of the biodiesel, the condition of storage and the degree of unsaturation of biodiesel-FAMEs and can be improved by the addition of antioxidants^41^ (Islam).^39^ OS was calculated according to the equation:
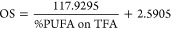
6

The saponification value (SV; mgKOH·g^–1^) and iodine value (IV; gI_2_·100g^–1^) of fat were estimated according to the following equations:
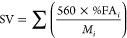
7
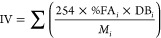
8where %FA*_i_* is the percentage of each FAME, DB_*i*_, and *M*_*i*_ are respectively the double bonds and molecular weight of the *i*th fatty acid.

The cetane number (CN) was calculated according to the following equation:
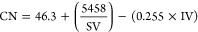
9where SV and IV are, respectively, saponification and iodine value.

Estimation of high heating value (HHV; MJ·kg^1–^), kinetic viscosity (KV; mm^2^·s^–1^), and density (ρ; g·cm^–3^) was based on the following equations:
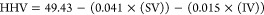
10
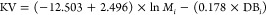
11
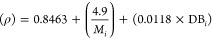
12

## Results and Discussion

### Heterotrophic Growth, Biomass, and Lipid Production of *Cyclotella cryptica*

**Figure 1:**
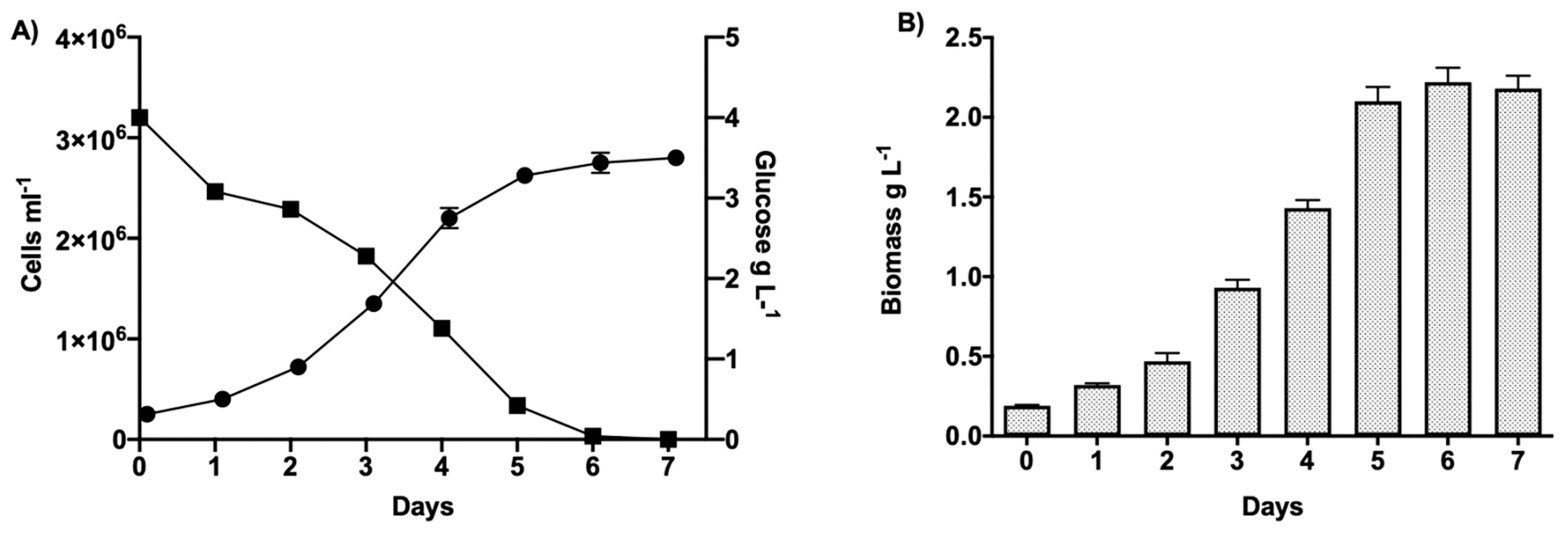
Cultivation of *C. cryptica* under heterotrophic conditions with 4 g L^–1^ glucose, C/N = 30, C/P = 525 and Si/C = 0.02. (A) Growth curve (circles, cells mL^–1^, left Y axis) and glucose consumption (square, g L^–1^, right *Y* axis); (B) biomass production (g L^–1^). Data are expressed as mean ± SD, *n* = 3.

The marine diatom *C. cryptica* was grown in the dark in 10 L carboys with glucose as sole organic carbon source at a concentration of 4 g·L^–1^ with an inoculum of 2.5 × 10^5^ cell mL^–1^. A sufficient macronutrient regime was guaranteed by a silicon-to-carbon (Si/C) ratio of 0.02, a carbon-to-phosphorus (C/P) ratio of 525, and a carbon-to-nitrogen (C/N) ratio of 30 in agreement with previous studies suggesting an optimal C/N ratio exceeding 20 to enhance lipid production in microalgae.^34,42,43^ As shown in Figure 1A, the growth rate indicated a high division rate with a doubling time of 1.5 ± 0.1 days and without an acclimation lag from autotrophic to heterotrophic conditions. Glucose measurements in the medium by proton NMR (Figure S1) showed complete consumption after 7 days, which correlated with the establishment of the stationary phase at a final cell density of 2.8 × 10^6^ cells mL^–1^ after 6 days. In the same days, the biomass production followed, increasing steadily along the growth curve and reaching a final concentration of 2.2 ± 0.09 g L^–1^ (Figure 1B).

Previous studies of biomass content from heterotrophic cultivation of microalgae have shown high variability in yields ranging between 0.2 to 109 g L^–1^ depending on glucose input, cell density and cultivation methods.^44,45^ On the other hand, except for few examples, sugar-to-biomass conversion of microalgae under heterotrophic conditions is generally below 0.5 g biomass per g glucose.^25^ The theoretical maximum yield for the conversion of glucose to biomass is 0.66 g biomass per g glucose in consideration of the loss of 1/3 of carbon as CO_2_ produced by decarboxylation of glycolytic pyruvate.^17^ In this study, we found that the glucose conversion in the biomass was 0.55 g of biomass per gram of glucose, which is more than 80% of the theoretical value and represents a promising result from the perspective of further implementations.

**Figure 2:**
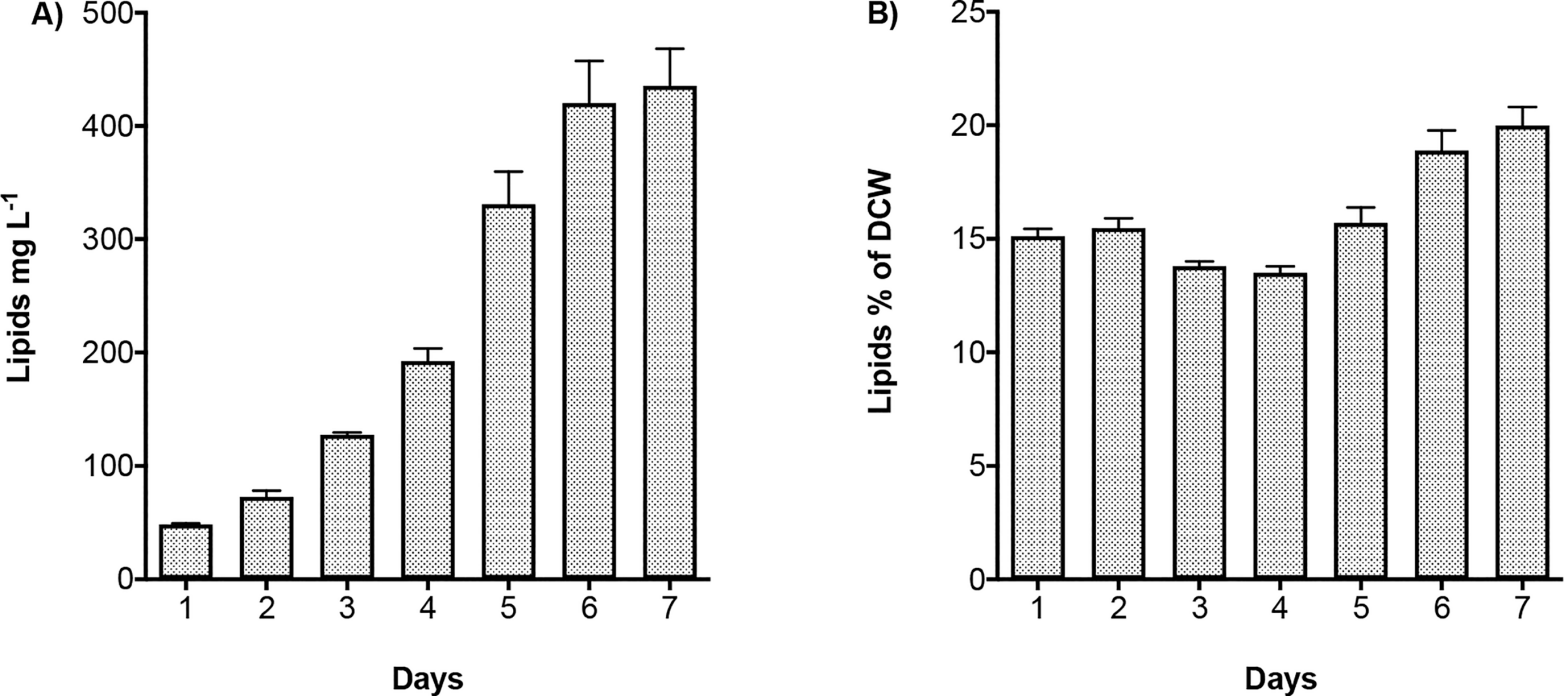
(A) Lipid production (mg L^–1^) and (B) lipid content (% of Dry Cell Weight) in *C. cryptica* grown under the heterotrophic conditions described in the legend of Figure 1. Data are expressed as mean ± SD, *n* = 3.

Total lipid content was consistent with the increase of the biomass and cell growth and reached the highest level of 435 ± 32.6 mg L^–1^ after 6 days (Figure 2A). Linear regression analysis supported a clear correlation of lipid content with cell number and biomass dry weight (Figure S2). Total lipids represented about 13–15% of the total biomass and showed a slight increase up to 20% only after 7 days in parallel with glucose consumption (Figure 2B).

Lipid accumulation in oleaginous microalgae depends on various factors, including strain physiology, harvest timing, nutritional regime, and cultivation methods.^20,44,46^ In heterotrophic cultures, the type and concentration of organic carbon sources as well as C/N ratio are the most critical factors influencing lipid content.^47^ The definition of these parameters was beyond the aim of the present study, but it is conceivable that a fine-tuning of the culture conditions could lead to a significant increase in lipids that we estimate could be up to 40–50% of total dry biomass.^43^

### Lipid Composition

**Figure 3:**
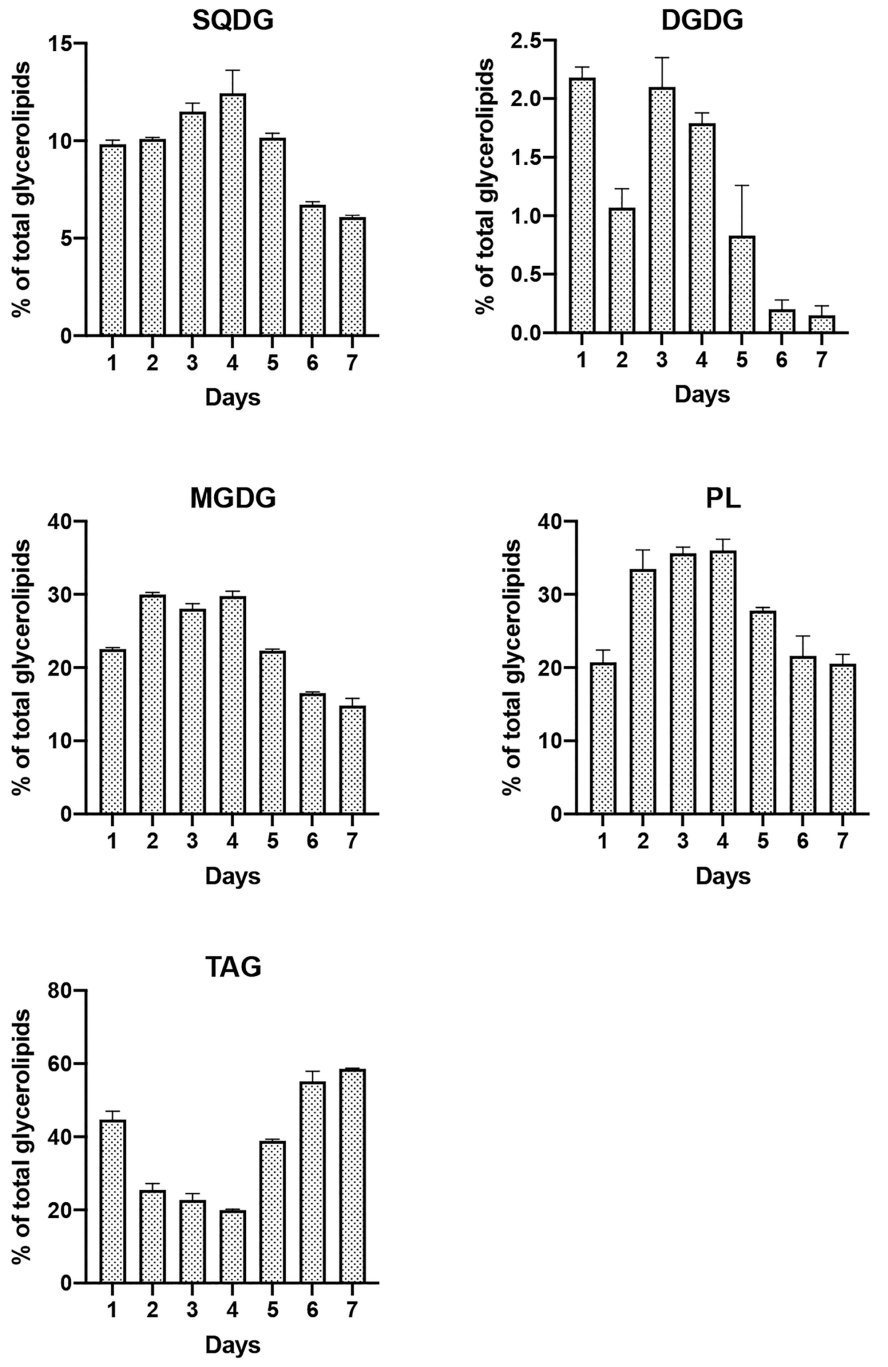
Glycerolipid composition assessed by proton NMR with the ERETIC method. Content is expressed as a percentage of total glycerolipids: TAG, triacylglycerides; MGDG, monogalactosyldiacylglycerols; DGDG, digalactosyldiacylglycerols; SQDG, sulfoquinovosyldiacylglycerols; PL, phospholipids. Data are presented as mean ± SD, *n* = 3

Distribution of glycerolipids was evaluated by the ERETIC method through integration of the diagnostic signals in the proton NMR spectra.^37^ Glycerolipids were mainly featured by triacylglycerols (TAG) (20–60%) and polar lipids, including phospholipids (PL) (20–36%) and glycolipids (GL) (21–44%) (Figure 3). This last group was composed of the plastid membrane components, namely, monogalactosyldiacylglycerols (MGDG) (14–30%), digalactosyldiacylglycerols (DGDG) (0.1–2.2%) and sulfoquinovosyldiacylglycerols (SQDG) (6–12%). Glycerolipid composition was subject to variation along the growth curve, with TAG accumulation accounting for nearly 60% of the extracts in the stationary phase. The increase in TAG was at the expense of GL which halved after 7 days whereas the effect on PL was slightly less. The levels of TAG were always greater than 20% in the biomass and the accumulation in the stationary phase was likely related to the shortage of glucose after 5 days.

This is clearly an important aspect to tune TAG production in heterotrophy and resembles the biochemical mechanisms that are triggered by nutrient depletion in autotrophic cultures.^20,48,49^ In reference to this, it has been reported that TAG production in *C. cryptica* can be increased from 5% to 60% by starvation of N and/or Si in photoautotrophic conditions.^50^ Although glycolipids and phospholipids are saponifiable, a high quantity of neutral lipids such as TAG increases transesterification velocity and FAME yields during the biodiesel production process.^51^ It is also worth noting that free fatty acids (FFA) were not detected along the growth curve, suggesting the presence of healthy cells and the absence of hydrolytic processes that occur in senescent cultures.^52,53^ Although FFA content between 0 and 70% has been reported, microalgae oil usually shows high levels of FFA which are considered a technical issue in conventional transesterification processes to biodiesel. The high FFA content is the result of hydrolytic reactions, both enzymatic and spontaneous, which depend on diverse factors, such as the microalgae genotype, temperature and time of harvesting, dehydration techniques, and biomass storage.^53−55^

### Repeated-Batch Process: Constant Cycle Repetitions

**Figure 4:**
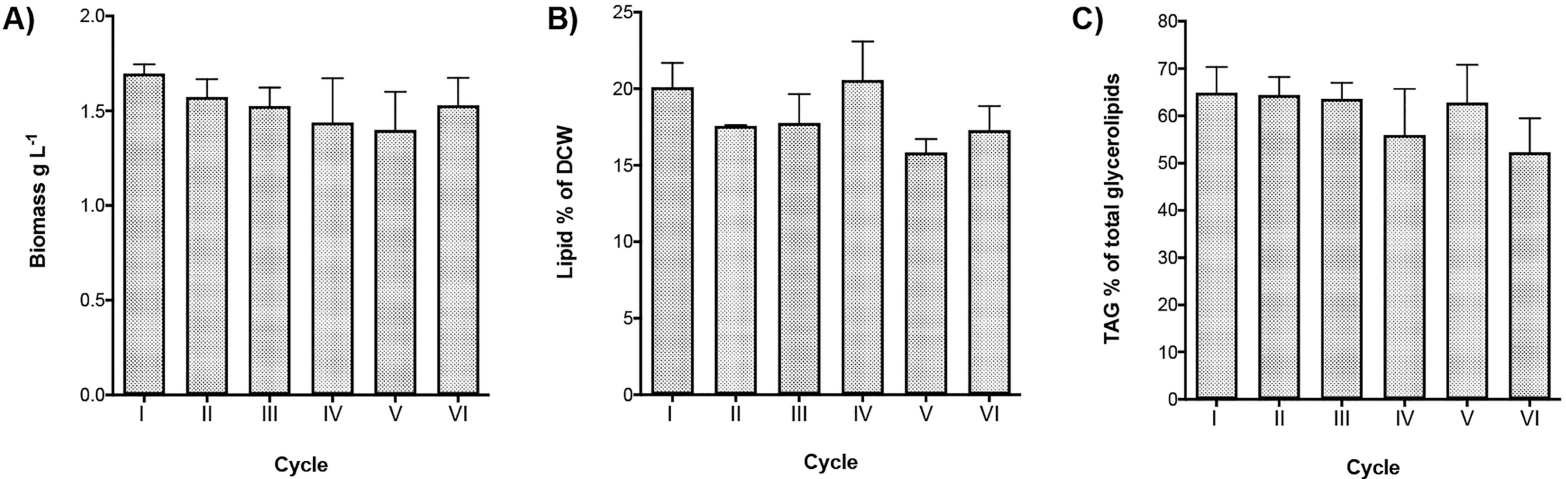
Biomass and lipid production for the 6 cycles (I–VI) of a repeated-batch process of *C. cryptica* under the heterotrophic conditions described in the legend of Figure 1 over 42 days. (A) Biomass production, expressed as g L^–1^ culture; (B) lipid content, expressed as % of Dry Cell Weight (DCW); (C) triglyceride (TAG) content expressed as percentage of total glycerolipids. Data are referred to the final point of each cycle after 7 days of cultivation per cycle. Data are expressed as mean ± SD, *n* = 6.

Repeated batch culture of *C. cryptica* in the dark was performed in consecutive cycles at regular 7-day intervals by using six 10L carboys per cycle (total culture volume of 60 L). At the end of each cycle (day 7), 90% of the batch was harvested, while the remaining 10% was used as seed culture and diluted with fresh medium at a ratio of 1:9 (v/v) in each carboy (Figure S3). The whole process was repeated for 6 cycles in 42 days. During this time frame, biomass production ranged from 1.7 to 1.4 g L^–1^ with a median value of 1.5 ± 0.1 g L^–1^ (Figure 4A). Considering biomass production, sugar-to-biomass conversion was between 0.37 and 0.45, with a median value of 0.4. Lipid content, indicated as % of dry cell weight, varied in a range from 16 to 21%, with a median value of 18% (Figure 4B).

Glycerolipid composition was not significantly affected by the repeated-batch production (not shown), thus supporting the stability of *C. cryptica* lipid metabolism under heterotrophic conditions. TAG was the most abundant pool and represented 54–65% of total glycerolipids (Figure 4C), with an average yield of 0.11 g TAG/g biomass. GL remained below 20%, whereas PL showed a higher variability ranging from 16 to 26% (Figure S4). Negligible levels of FFA were detected in the lipid extracts. This result is interesting because it reduces the risk of soap formation affecting the downstream biomass fraction and the partial consumption of the catalyst used for biodiesel production.^56^ The data collected at the end of each cycle corroborated the high reproducibility and stability of the entire process. Notably, it was not necessary to use antibiotics to control the bacterial contamination. This result was rather unexpected but important in view of the reduction of the operational costs and highlighted the robustness of *C. cryptica* strain for the heterothrophic production of biomass.

### Fatty Acid Composition

It is well-known that the fatty acid composition of microalgae is highly impaired by growth conditions including trophic factors, nutrients, temperature, and light intensities.^24,48,57^ For this reason, the total fatty acid profile of *C. cryptica* was assessed by GCMS after transesterification of lipid extracts obtained at the end of each cycle of the repeated-batch process. The average composition of the fatty acids methyl esters (FAME) showed the presence of palmitic acid (16:0, ∼26%), palmitoleic acid acid (16:1 ω-7, ∼57%), and eicosapentaenoic acid (20:5 ω-3, ∼12%). These three species accounted for 95% of the total fatty acids (Table 1) and affected the overall levels of saturated fatty acids (SFA), monounsaturated fatty acids (MUFA), and polyunsaturated fatty acids (PUFA).

**Table 1:** Fatty Acid Composition of Common Plant Oils (Palm, Rapeseed, and Soybean) and *C. cryptica*^a^

fatty acids	*Cyclotella cryptica*	palm	rapeseed	soybean
12:0	0	0	0	0
14:0	2.1 ± 0.3	1.1	0	0.1
16:1 ω-7	56.6 ± 4.2	0.2	0.1	0.2
16:0	25.6 ± 3.5	42.5	4.2	11.6
18:3 ω-3	0	0.3	8.4	5.9
18:2 ω-6	0.3 ± 0.1	9.5	21.5	53.8
18:1 ω-9	3.6 ± 1.2	41.3	59.5	23.7
20:5 ω-3	11.8 ± 1.0	0	0	0
				
SFA (%TFA)	27.8	43.60	4.20	11.70
MUFA (%TFA)	60.2	41.50	59.60	23.90
PUFA (%TFA)	11.9	9.80	29.90	59.70

a Grown by a repeated-batch process for 42 days (six cycles) under the heterotrophic conditions described in the legend of Figure 1. Data are expressed as means ± SD, *n* = 36. SFA = Saturated Fatty Acids; MUFA = MonoUnsaturated Fatty Acids; PUFA = PolyUnsaturated Fatty Acids, TFA= Total Fatty Acids.

According to Cupo et al.,^34^ heterotrophic conditions led to the loss of the typical plastid PUFAs of diatoms, namely, 16:2 ω-4, 16:3 ω-4, and 16:4 ω-1, which were replaced by 16:1 ω- 7. Eicosapentaenoic acid (20:5 ω-3) did not significantly change in comparison to previous data with autotrophic cultures of the same strain. Heterotrophic growth of *C. cryptica* resulted in low levels of PUFA (<12%) compared to other microalgae (e.g., *Chlorella* species) which, cultivated in both autotrophic and heterotrophic conditions, show levels of PUFAs generally accounting for 40% of the total fatty acids due to the presence of linolenic acid (18:3 ω-3) and linoleic acid (18:2 ω-6).^26,58^ Palmitoleic acid (16:1 ω-7) is the main MUFA in *C. cryptica*, which replaced oleic acid (18:1 ω-9) commonly present in other microalgae.

### Estimation of *C. cryptica* Biomass as a Feedstock for Sustainable Biodiesel Production

The composition and chemical structures of fatty acids determine the physio-chemical properties of biodiesel and affects the compliance with international standard specifications.^39,59^ Soybean, palm and rapeseed are the main feedstocks for the world production of biodiesel.^60,61^ These common vegetable oils share the presence of the same four major chemical species, namely, palmitic acid (16:0), oleic acid (18:1 ω-9), linoleic acid (18:2 ω-6), and linolenic acid (18:3 ω-3).^60^ However, the compositional profiles can vary significantly as palm oil shows a high percentage of SFA and MUFA, while soybean and rapeseed show a higher concentration in C_18_–PUFAs (Table 1).^40^ In order to evaluate the potential of *C. cryptica* as an alternative source of vegetable biodiesel, Table 2 reports a comparative analysis of the biodiesel properties from these plants and *C. cryptica* on the basis of the fatty composition reported in the literature or obtained in the present study.

**Table 2:** Estimation of Biodiesel Properties Based on the Fatty Acid Profile of *C. cryptica* Grown under Heterotrophic Conditions^a^

biodiesel properties	unit	*Cyclotella cryptica*	palm	rapeseed	soybean	ASTM D6751	EN 14214
long-chain saturation factor LCSF		2.56					
oxidative stability, 110 °C	h	12.3	13.37		3.8	≥3	≥6
density	g/cm^3^	0.96	0.86	0.91			0.86–0.90
cold filter plugging point	°C	–8.4	9	–12	–4		
cetane number		41.5	61.9	53.7	51.3	≥47	≥51
kinematic viscosity	mm^2^/s	3.5	4.61	4.5	4.26	1.9–6.0	3.5–5
iodine value	g I_2_/100 g	118.1	54	116.1	125.5		≥120
high heating value	MJ/kg	38.8	40.6	41.1	39.7		

a The diatom product was evaluated against the most common plant oils (palm, rapessed and soybean) and according to its compliance with the international standards specifications (ASTM D6751 and EN 14214).

It is generally accepted that biodiesel should contain high concentrations of SFA and MUFA together with low concentrations of PUFA and long-chain fatty acids, although chemical characteristics of fatty acids can have contrasting effects on the properties of the fuel.^62^ Among the factors that influence the use of an oil as a fuel blendstock, the most important include cetane number (CN), cold filter plugging point (CFPP), long-chain saturation factor (LCSF), viscosity, oxidative stability, and density.^63,64^

Long-chain saturation factor (LCSF) measures the influence of the saturated fatty acids on the oil quality, in particular, low temperature or cold flow performance of biodiesel. The oil obtained from *C. cryptica* gave a value of 2.56 mainly due to the concentration of palmitic acid. The international standard specifications do not establish a limit for LCSF but this parameter is closely linked to CN, CFPP, and viscosity. For instance, a high LCSF number gives a high cetane number (CN) value and reduces NO_*x*_ emissions.^65^

CN determines the ignition properties of the fuel and decreases with increasing degree of unsaturation and length of fatty acid chains.^56^ The low CN for *C. cryptica* in comparison to those of the plant oils is due to the lower concentration of PUFA and the longer chain and higher unsaturation of PUFA, mainly due to eicosapentaenoic acid. The cold filter plugging point (CFPP) is the lowest temperature (°C) at which biodiesel flows through a standardized filter device for a specific period of time. This parameter is a measurement of the performance during the cold weather.^66,67^*C. cryptica* oil was estimated to have a value of −8.4, which is between rapeseed and soybean, thus suggesting that the biodiesel from the diatom could operate under low-temperature conditions. Oxidation stability depends on the unsaturation degree of fatty acids and is one of the major problems when using biodiesel. For biodiesel samples, a minimum Rancimat induction time of 3 and 6 h is defined in ASTM6751 and EN 14214, respectively. Biodiesel obtained from *C. cryptica* has an oxidative stability of 12 h, thus satisfying the criteria stipulated by both regulatory entities. High kinematic viscosity can lead to mechanical problems, such as engine deposits. The viscosity increases with increasing molecular weights of FAs and decreases with an increasing number of double bonds. The predicted value for the kinematic viscosity of *C. cryptica* is 3.5 mm^2^/s, which complies with the range established by ASTM D6751 (1.9–6 mm^2^/s) and EN 14214 (3.5–5 mm^2^/s). Fuel density is a critical property impacting engine performance, such as the energy content of the combustion chamber and the air–fuel ratio. The high unsaturation degree of the fatty acids results in a high density, while the long chains reduce the density of the derived biodiesel.^60^ The predicted value of the oil produced from *C. cryptica* is 0.96 g cm^–3^, slightly higher than the limit imposed by the European legislation of 0.860–0.900 g cm^–3^ at 15 °C.

In conclusion, biodiesel properties derived from biodiesel of *C. cryptica* grown in heterotrophic conditions comply with or are very near the official specifications required by American and European regulations. We estimated a good oxidative stability compared to the biodiesel derived from the most common plant feedstocks. In particular, it was comparable to palm oil and better than soybean and rapeseed oils that have a high percentage of PUFA (e.g., oxidation stability, iodine value, high heating value). Regarding to the performance at low temperatures (LCSF, CFPP, KV), the diatom product is suggested to perform better than the biodiesel derived by palm oil. In consideration of the oil quality, these data support the idea that the repeated-batch cultivation of *C. cryptica* under heterotrophic conditions can be a suitable process for biodiesel production. Further studies are necessary to further fine-tuning of the cultivation parameters to increase total biomass and lipid productivity, as well as to valorize sugar-based biowaste and increase the profitability of the entire process.

## Supporting Information Available

^1^H NMR spectra of glucose-containing medium; linear regression analysis of lipids against cell number and biomass; picture of 10L heterotrophic cultures; detailed glycerolipid distribution in different cycles of repeated batch process (PDF)
