# Supplementary material for: Potential of Lipid Biosynthesis under Heterotrophy in the Marine Diatom Cyclotella cryptica
Source: ACS Sustain Chem Eng. 2023 Dec 4;11(50):17607–15. doi: 10.1021/acssuschemeng.3c02542 (PMC10731643; doi:10.1021/acssuschemeng.3c02542)

Supporting Information for

Potential of lipid biosynthesis under heterotrophy in the marine diatom *Cyclotella cryptica*

Salvatore Morra [a,‡], Mariamichela Lanzilli [a,‡], Angela Grazioso [a,‡], Adelaide Cupo [a], Simone Landi [b], Genoveffa Nuzzo [a], Daniela Castiglia [a], Carmela Gallo [a], Emiliano Manzo [a], Angelo Fontana [a,b] and Giuliana d'Ippolito [a,]*

[a)] National Research Council (CNR), Institute of Biomolecular Chemistry (ICB), Via Campi Flegrei 34, 80078 Pozzuoli, Naples, Italy.

[b)] Department of Biology, University of Naples "Federico II", Via Cinthia, I-80126 Napoli, Italy.

** Corresponding author: gdippolito@icb.cnr.it – Phone:+39 081-8675096*

‡ *S.M, M.L. and A.G. contributed equally to this paper.*

Pages:5

Figure: S1, S2, S3 and S4

FIGURE S1: ^{1}H-NMR spectrum of the supernatant of the culture medium containing 4 gL-1 of glucose, after cell centrifugation. The diagnostic peak, centered at 3.24 ppm, refers to the signal of protons at C-2 of ß-anomer of glucose.

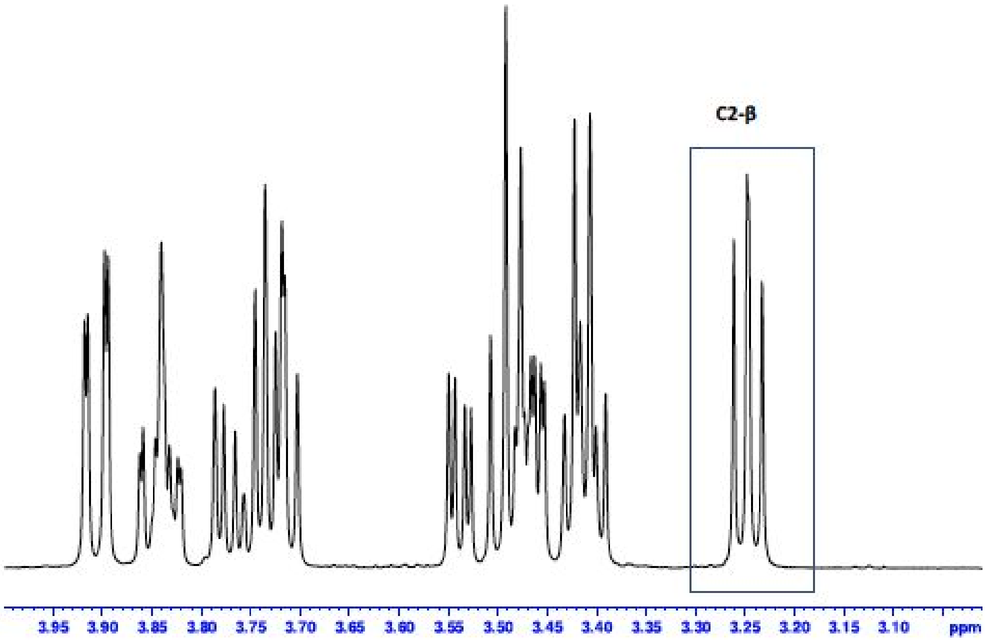

FIGURE S2: Linear regression analysis of lipids against number of cells and biomass at day 7

Variable #1	Variable #2	*r*	r^2	*F sign*	*Equation*
Lipid	Number of Cell	0.94	0.90	0.044	y = 0.0001x – 27.063
Lipid	Biomass	0.97	0.95	3,43E-05	y = 185.63x – 19.672

FIGURE S3: Heterotrophic cultures of *Cyclotella cryptica* in 10L carboys (A).

(A)

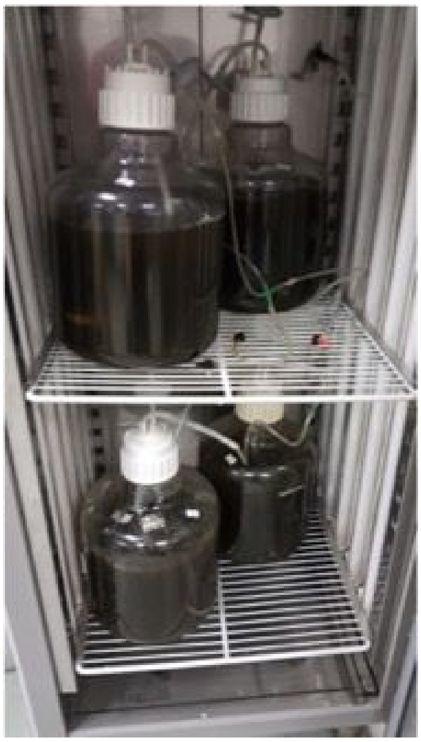

FIGURE S4: *Glycerolipid distribution assessed by ^{1}H-NMR at the end of each cycle for the 6 cycles (I-VI) of repeated batch process of C. cryptica under heterotrophic conditions. Results are expressed as percentage of total glycerolipids. TAG triacylglycerides, MGDG monogalactosyldiacylglycerol, DGDG digalactosyldiacylglycerol, SQDG sulfoquinovosyldiacylglycerol, PL phospholipids*
Data are presented as mean ± SD, n = 3

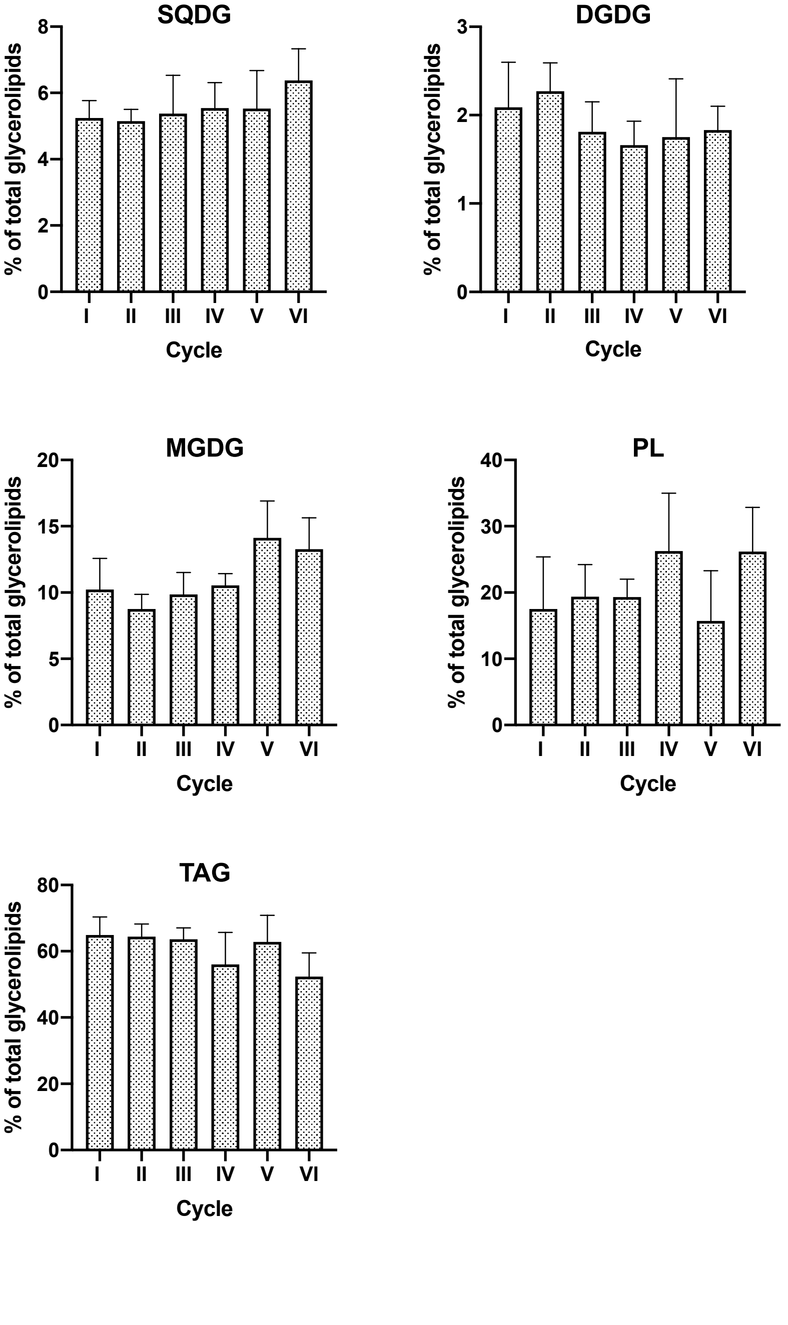

Supplement: Supplementary file 1 — sc3c02542_si_001.pdf [file sc3c02542_si_001.pdf]
